# The Regulation of JNK Signaling Pathways in Cell Death through the Interplay with Mitochondrial SAB and Upstream Post-Translational Effects

**DOI:** 10.3390/ijms19113657

**Published:** 2018-11-20

**Authors:** Sanda Win, Tin Aung Than, Neil Kaplowitz

**Affiliations:** Division of Gastrointestinal and Liver Disease, Department of Medicine, Keck School of Medicine, University of Southern California, Los Angeles, CA 90033, USA; swin@usc.edu (S.W.); tthan@usc.edu (T.A.T.)

**Keywords:** reactive oxygen species, PTPN6, SRC, DOK4, p38, MKK4, MKK7, p53, DUSP1, SIRT2

## Abstract

c-Jun-N-terminal kinase (JNK) activity plays a critical role in modulating cell death, which depends on the level and duration of JNK activation. The kinase cascade from MAPkinase kinase kinase (MAP3K) to MAPkinase kinase (MAP2K) to MAPKinase (MAPK) can be regulated by a number of direct and indirect post-transcriptional modifications, including acetylation, ubiquitination, phosphorylation, and their reversals. Recently, a JNK-mitochondrial SH3-domain binding protein 5 (SH3BP5/SAB)-ROS activation loop has been elucidated, which is required to sustain JNK activity. Importantly, the level of SAB expression in the outer membrane of mitochondria is a major determinant of the set-point for sustained JNK activation. SAB is a docking protein and substrate for JNK, leading to an intramitochondrial signal transduction pathway, which impairs electron transport and promotes reactive oxygen species (ROS) release to sustain the MAPK cascade.

## 1. Introduction 

c-JUN-N-terminal kinase (JNK) is a critical mediator of physiological and pathological responses. An upstream MAP kinase signaling cascade from dual specificity MAP3K (e.g., ASK1) and MAP2K (MKK4/7) to serine/threonine MAPK (e.g., JNK, p38) mediate both the initiation of activation of JNK and its sustained activation [1,2,3]. There is a critical distinction between transient activation of the signaling pathway lasting minutes versus sustained activation lasting hours or more [4]. In this focused review, we will discuss the recent identification of JNK-mitochondrial SAB (SH3BP5)-ROS activation loop in sustaining the MAP kinase cascade, leading to pathological consequences within the context of our interest in liver disease. In addition, we will discuss a variety of factors that regulate or modulate the MAPK pathway from upstream MAP3K, such as apoptosis signal-regulating kinase 1 (ASK1) and mixed lineage kinase 2/3 (MLK2/3), to downstream JNK, SAB, and mitochondrial ROS. This review highlights the pathophysiological mechanism of sustained activation of JNK through JNK-activation loop and opens possible pharmacological interventions for therapeutic targets in the liver, heart, and brain, where SAB has been shown to have an important role. 

## 2. JNK-SAB-ROS Activation Loop 

Interest in the role of JNK signaling in liver injury began nearly 20 years ago with the identification of the protection of cultured mouse hepatocytes from acetaminophen (APAP)-induced necrosis by a JNK inhibitor (SP600125) [5]. This was further supported by the discovery of protective effect in in vivo JNK1 and 2 double knockdown using antisense oligonucleotides [6]. In this work and later in other in vivo and in vitro models of JNK-dependent liver apoptosis (e.g., TNF/D-galactosamine [TNF/GalN], tunicamycin-induced endoplasmic reticulum (ER) stress, and lipoapoptosis), a key important finding, i.e., the association of P-JNK with mitochondria, was discovered. Then, the binding target of P-JNK on mitochondria and the significance of this interaction were uncovered [7,8,9]. This seemed particularly relevant as earlier works had suggested that mitochondrial ROS played an important role in the sustained activation of JNK and that antioxidants could protect against sustained JNK activation and apoptosis in response to TNF [8,10]. 

One prior publication by Wiltshire et al. had identified a mitochondrial outer membrane protein—SAB (SH3BP5)—as a target of P-JNK binding and substrate for JNK phosphorylation [11]. These studies were performed in chicken embryotic fibroblast cells, and the functional consequences of the interaction were not further explored [11]. Then, SAB was identified exclusively in the outer membrane of mitochondria in liver [12]. SAB and P-JNK coimmunoprecipitated shortly after toxic stress from APAP prior to overt liver injury [7,13]. The finding was further supported by adenoviral sh-SAB (versus sh-lacZ control)-mediated depletion of SAB in liver. Knockdown of SAB resulted in inhibition of sustained JNK activation and translocation to mitochondria in all the models of JNK-dependent toxicity (APAP, TNF/GalN, ER stress, palmitic acid lipotoxicity) in vivo or in vitro. This was accompanied by striking protection against cell death [7,8,9]. More recently, these findings have been fully confirmed using hepatocyte-specific inducible knockout of SAB in two-month-old mice either after crossing *SAB^fl/fl^* mice with transgenic tamoxifen inducible *alb-CRE^+/−^* mice followed by tamoxifen feeding or by injection of *SAB^fl/fl^* mice with hepatocyte-targeted CRE viral vectors (adeno-alb-CRE or AAV8-TBG-CRE) [12]. 

Another important issue to address is the effect of the interaction of P-JNK with SAB on mitochondrial function and ROS production. Using isolated normal liver mitochondria, recombinant P-JNK1 and/or 2 in the presence of ATP was shown to lead to inhibition of oxidative phosphorylation and maximum respiratory capacity [12]. This effect was not observed in the absence of ATP, suggesting that phosphorylation of SAB was required. Furthermore, this effect was accompanied by enhanced O_2_ production in MitoSOX-loaded mitochondria [8,14]. The effect of P-JNK + ATP was absent in liver mitochondria from SAB knockout mice and was inhibited by a peptide corresponding to the JNK docking site of SAB, which blocked the interaction of JNK and SAB [8,12]. 

The topology of SAB can be defined using c- and n-terminal-targeted antisera. The short c-terminus faces the cytoplasm and contains a JNK kinase interaction motif (KIM), which is the docking site. The longer N-terminus faces the intermembrane space [12]. Since there is no evidence that JNK enters the mitochondria, the question relates to how the interaction of P-JNK with SAB and its phosphorylation on the external face lead to impairment of mitochondrial bioenergetics. The mechanism of JNK-SAB-mediated impairment of mitochondrial respiration has been explored [12]. Tyrosine-protein kinase c-SRC, mainly in the P-419-SRC active state, has been shown inside mitochondria of liver and neurons and is required to maintain the function of the electron transport chain. SRC kinase inhibitors reproduce the same effect as P-JNK/ATP on isolated mitochondria. When mitochondria were exposed to P-JNK/ATP, rapid dephosphorylation of P-SRC was observed, but this did not occur in mitochondria from SAB knockout liver and was also inhibited by the KIM blocking peptide [8,9,12]. Furthermore, inactivation (dephosphorylation) of SRC occurred in liver mitochondria after in vivo treatment with APAP or TNF/GalN. Inactivation of SRC was inhibited in isolated mitochondria after treatment with vanadate, which blocked the effect of P-JNK/ATP on mitochondrial respiration. The study indicated that a phosphotyrosine phosphatase (PTP) was responsible for mediating the effect and that intramitochondrial protein tyrosine phosphatase non-receptor type 6 (PTPN6/SHP1) was responsible for inactivation of SRC when JNK interacted with SAB on mitochondrial outer membrane. Mitochondria isolated from PTPN6-depleted mice were resistance to the effects of P-JNK/ATP on P-SRC and mitochondria respiration [12].

**Figure 1:**
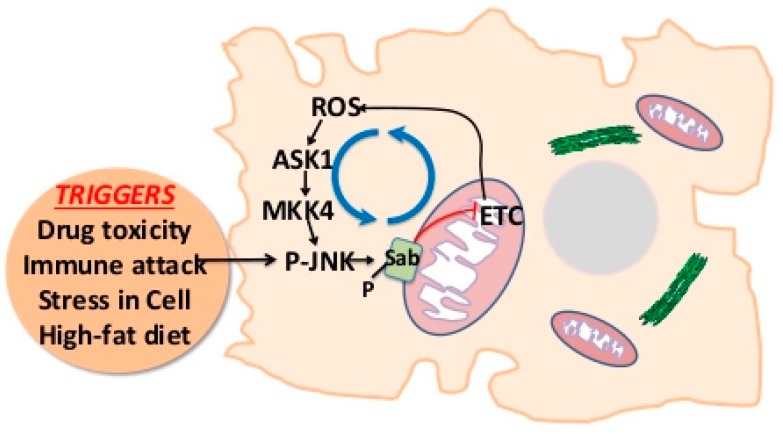
P-JNK-SAB-mitochondria-ROS-mediated JNK activation loop. JNK activation is triggered by physical and chemical stress, including alterations in nutrients, growth factors, cytokines, extracellular matrix, DNA damage, drugs, and toxins. Activated JNK translocates to mitochondria and interacts with SAB, leading to a sequence of events, i.e., inhibition of intramitochondrial c-SRC activity and mitochondrial electron transport chain and thus release of ROS, which further activates ASK1, MKK4/7, and JNK. P-JNK-SAB-ROS activation loop drives sustained JNK activation, and cell death occurs. Black arrows indicate activation. Blue circulating arrows indicate vicious cycle. Red “T” arrow indicates inhibition of electron transport chain (ETC).

Mitochondrial SRC associates with docking protein 4 (DOK4), a kinase and PTP docking protein [15], and DOK4 participates in the JNK activation of intramitochondrial signaling pathway [12]. DOK4 is found exclusively in the mitochondria fraction and is associated with the inner membrane but accessible to the intermembrane space. Knockdown of DOK4 in vivo protected isolated mitochondria from the effect of P-JNK/ATP and protected against liver injury and sustained JNK activation. The effect of DOK4 knockdown was analogous to the knockout of upstream SAB or knockdown of PTPN6. Careful mitochondrial subfractionation and immunoprecipitation studies have revealed that under basal conditions, SAB is in the outer membrane and bound SHP1 (on the intermembrane face), while DOK4 and P-SRC are on the inner membrane. Following toxic stress and JNK activation, PTPN6 coimmunoprecipitation with SAB decreases in the outer membrane fraction and binding to SRC/DOK4 (coimmunoprecipitation) increases on the inner membrane. Thus, when SAB is phosphorylated by P-JNK/ATP on the cytoplasmic face, PTPN6 is released and interacts with P-SRC, dephosphorylating (inactivating) SRC in a DOK4-dependent fashion. Thus, DOK4 appears to serve as a platform, which is required for the interaction of PTPN6 and P-SRC. PTPN6 associated with SAB is inactive (nonphosphorylated); however, when associated with P-SRC, it is phosphoactivated by SRC, which then leads to dephosphorylation of SRC. Inactivation of SRC leads to impaired mitochondrial respiration and increased ROS release from mitochondria. ROS then activates ASK1, and possibly MLK2/3, which sustains activation of MKK4/7, leading to sustained JNK activation (Figure 1). ROS oxidize thioredoxin, relieving ASK1 of inhibition of dimerization and allowing self-activation of ASK1. ROS also activate SRC at or near the plasma membrane, which then activates MLK2/3. These MAP3 kinases then activate MAP2 kinases, which activate JNK.

The duration and degree of sustained JNK activation mediates many consequences, both through transcriptional regulation by AP-1 targets that modulate expression of many genes involved in proliferation as well as inflammation (cytokines and chemokines), metabolic gene dysregulation e.g., gene repressors, such as nuclear receptor corepressor 1 (NCOR1) action on peroxisome proliferator activated receptor alpha (PPARα) and thioredoxin-disufide reductase (TR), or through direct activation of proapoptotic BH3 family members and inhibition of antiapoptotic Bcl2 family members (see Reference [3] for review).

## 3. Modulation of JNK Activation Loop 

**Figure 2:**
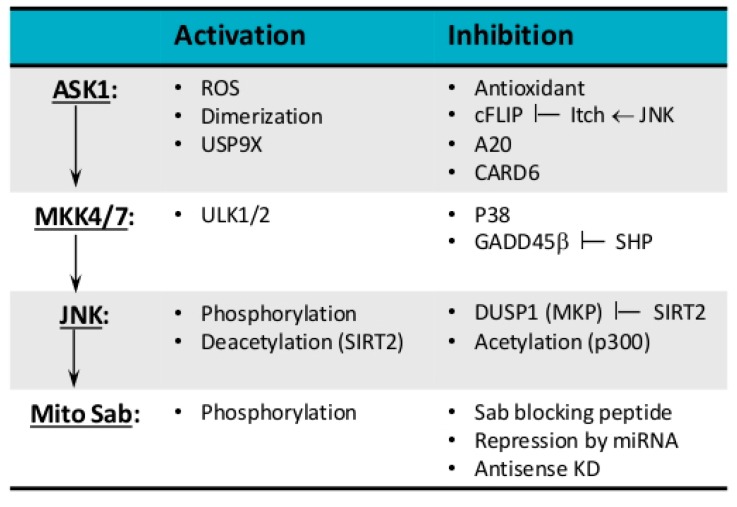
Modulation of JNK activation loop. JNK-SAB-ROS activation loop can be modulated at all level of MAP kinase cascade through phosphorylation by upstream kinase, dephosphorylation by phosphatase, acetylation by sirtuins, deacetylation by p300, protein stabilization by deubiquitinating enzymes, and protein degradation by ubiquitinating enzymes.

**Figure 3:**
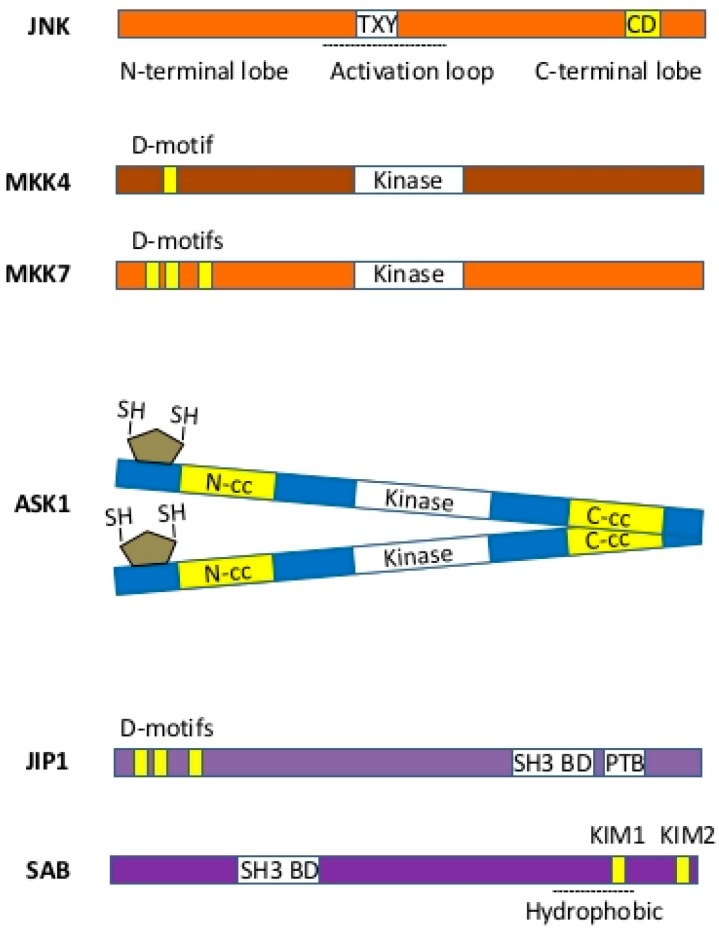
Schematic diagram of molecules involved in JNK activation loop. JNK is activated by dual threonine–tyrosine phosphorylation at (TXY) located within activation loop (indicated as black dotted line) by MKK4/7 through interaction with common docking site (CD) of JNK and docking motif (D-motif; JNK binding site indicated by yellow bars) of MKK4/7. CD of JNK is also shared with JIP and SAB which has one hydrophobic transmembrane spanning domain (indicated as dotted line). Two JNK binding sites on SAB are noted as KIM1 and KIM2 (indicated by yellow bars). Dimerization of ASK1 through interaction of N-terminal coil-coil domains (N-cc) and of C-terminal coil-coil (C-cc) doamins autoactivates ASK1. Removal of thioredoxin (indicated by gray polygon) by oxidation facilitates dimerization of ASK1.

MAP kinase cascade, which senses cellular and extracellular stress, conveys cellular response to regulate cell fate. The timing and duration of JNK activation determine whether cells proliferate or adapt to metabolic or toxic stress or undergo programmed cell death, such as apoptosis, necrosis, and possibly other forms of cell death. Thus, modulators of the JNK-SAB-ROS activation loop (Figure 2) and molecular structure of components in the loop (Figure 3) determine the duration of JNK activation and selectivity and specificity of JNK-mediated cellular responses. As the expression of isoforms of kinases, phosphatases, substrates, inhibitors, and scaffold proteins involved in the JNK activation loop are tissue and cell-type-specific, we will discuss the general principles of modulation of the loop. 

### 3.1. MAP Kinases—JNK and p38

JNK and p38 are activated by MKK4/7 via dual phosphorylation of a Thr-Pro-Tyr (TPY) motif of the activation loop (A-loop), which connects N-terminal and C-terminal lobes. The ATP binding site lies between the lobes [16]. One critical feature of JNK signaling is the use of its single common docking site (CD) to interact with JNK-binding domain (D-motif) of upstream MKKs, MAP kinase phosphatases, substrates, inhibitors, and scaffold proteins. Thus, JNK activity derives from the ensemble of interactomes that compete for the docking site on JNK. Therefore, reducing the level of a single substrate of MAPK can lead to decreased amounts of active MAPK due to loss of competition and greater access of phosphatases to allow graded response. The specificity of JNK signaling relies on hydrophobic φ-x-φ docking motif (D-motif) on JNK substrates and scaffold proteins, such as c-Jun, ATF2, JIP, and SAB, and their subcellular localization. Preferences for D-motifs on different JNK substrates have been analyzed using 11-mer peptides derived from different substrates [17]. SAB peptide derived from KIM1 D-motif is similar to JIP1 sequence but has 21 folds lower affinity for P-JNK. This appears to be due to adjacent Pro residue. This means that small changes in D-motif and adjacent residues can profoundly impact JNK signaling. Indeed, JIP1 has higher affinity for JNK3 than ATF2 or SAB. Cytosolic JIP is a platform to bring together upstream kinases MKKs, MLKs, and Rac for JNK activation [18,19,20]. The depletion of JIP in MEF cells prevents fatty-acid-induced JNK activation [20]. JNK phosphorylates c-JUN on Ser63/73 and increases c-JUN-dependent transcription and cell proliferation [1], whereas JNK interaction with less affinity to SAB localized on mitochondria accounts for ROS generation, sustained MAP3K to JNK activation, and cell death [7,8,9,12,21]. Importantly, JNK phosphorylates p53 on Thr81 and stabilizes and confers its transcriptional activity [22], which could dampen expression of SAB and the JNK activation loop (see below). 

A distinct difference of p38 compared to JNK is the activation of downstream MAPK-activated protein kinase (MAPKAPKs), such as MK2/3, MNK1/2 [23]. p38 regulates MK2-mediated TNF-α and IL-6 production by promoting translation and/or stability of their mRNAs [24]. p38 modulation upregulates antioxidant response via NF-κB [25] and interferes with ROS produced by the JNK activation loop. p38-mediated MK2 activation also phosphorylates MDM2 on Ser157 and Ser166, resulting in MDM2 activation and degradation of p53 [26]. p53 expression and activity, contributed to by multiple signaling pathways [27,28,29], importantly regulates SAB expression and determines susceptibility to promoting the JNK activation loop (abstract, manuscript in preparation). The depletion of p53 or inhibition of p53 by pifithrin leads to higher P-JNK levels and more severe necrosis in acetaminophen-induced acute liver injury [30]. Recently, we uncovered an important regulatory role of p53 in SAB expression and thus the contribution of functional p53 in decreasing JNK-mediated cell death (see below). In addition, human rhabdomyosarcoma cells lacking functional p53 undergo rapid apoptotic cell death in mild cellular stress conditions, such as serum-free condition and inhibition of mTOR, through the ASK1-JNK activation-mediated pathway [31]. The mechanism may be by the upregulation of SAB expression in p53 deficiency, which we will discuss further below. This could be a potential therapeutic pathway to target treatment of functional p53-defective tumor cells.

Dual-specificity protein phosphatases (DUSP), which are also known as mitogen-activated protein kinase phosphatases (MKP), are a family of threonine-tyrosine dual-specificity phosphatases that dephosphorylate and inactivate MAPKs such as extracellular regulated MAP kinase (ERK), p38, and JNK in a context-dependent manner [1,32]. The highest levels of DUSP1 are observed in the heart, lungs, and liver. *DUSP1* or *DUSP5* KO mice were shown to exhibit increased JNK activity due to physiological levels of reactive oxygen species, supporting the role of phosphatase in the JNK activation loop [2,10]. Indeed, DUSP inhibition may be sufficient to induce prolonged activation of JNK following some stimuli. DUSPs localize in cytoplasm and nucleus, but mitochondrial localization or association has not been reported. Indeed, DUSP1-mediated dephosphorylation of JNK is sufficient to inhibit JNK-SAB-ROS activation loop because activation of JNK is required for translocation and interaction with mitochondrial SAB. In breast cancer, an inhibitor of DUSP1 is considered an adjuvant and/or neoadjuvant therapy by enhancing cell apoptosis. The involvement of p38 in modulation of JNK activation loop is more complex because p38/ERK upregulates DUSP1 expression through MAPK-activated protein kinase MSK [23].

In addition to phosphorylation and dephosphorylation, direct regulation of JNK activity also involves participation of acetylation/deacetylation. Cytosolic and nuclear shuttling SIRT2 deacetylates and activates JNK, whereas JNK is inhibited by p300-mediated acetylation. In addition, SIRT2 deacetylates and inhibits DUSP1 [33]. Thus, sirtuin activation occurring in excess nicotinamide adenine dinucleotide (NAD^+^), such as in nutrient excess or granulocyte-colony stimulating factor (G-CSF) signal transduction, changes the balance of JNK activation. However, JNK activation by sirtuin inducers [34] cannot reach the threshold level to cause sustained JNK activation and therefore does not favor cell death. In addition, sirtuin 1 (*SIRT1*) knockout mice were shown to exhibit less severe liver injury through preconditioned enhanced NF-κB response and dampening sustained JNK activation in the GalN/LPS-induced apoptotic cell death. However, acetaminophen-induced liver injury, which overrides NF-κB-mediated upregulation of antioxidant genes, is not protected [35]. Overall, the contribution of sirtuins in cell death pathways appears to be of minor importance. So far, the role of SIRT3, 4, and 5 in mitochondria have not been explored in the mechanism of JNK-SAB-ROS activation loop [36]. In summary, p38, p53, DUSP1, SIRT2, p300, NF-κB, Gadd45β, and SAB all have the potential to directly or indirectly modulate JNK activity in a complex, context-dependent fashion.

### 3.2. MKKs—MKK4 and MKK7

MKK4/7 interacts with JNK via D-motif and phosphorylates and activates JNK on JIP platform [37]. In MEF cells, MKK4 also activates p38. MKK7 contains three JNK docking D-motifs within its 100-amino acid regulatory domain. The second docking site of MKK7 binds to JNK via two alternative binding modes [38]. However, the significance and selectivity of the D-motif of MKK7 on JNK needs further exploration. Interestingly, Ser403 of MKK7 in HEK293 cells is phosphoactivated by serine/threonine-protein kinase ULK1/2 (ATG1/2), which is a downstream target of AKT/mTOR signaling pathway. The significance of this crosstalk in the sustained JNK activation loop in disease models requires further exploration as liver-specific deficiency of ULK1/2 in KO mice were shown to delay and partially protect liver injury from acetaminophen-induced hepatoxicity but not GalN/TNF liver injury [39]. Signalosomes, such as receptor complex in TNF-induced JNK activation and TRAFII-mediated JNK activation in ER stress, are important in integration of crosstalk, induction, amplification, and inhibition of the JNK activation loop [40]. The cytosolic JNK interacting protein (JIP) platform is crucial for initial activation of JNK in fatty acid but not in TNF-induced JNK activation [20]. SRC phosphorylation of JIP1 creates phosphotyrosine interaction motifs that bind the SH2 domains of SRC and the guanine nucleotide exchange factor VAV which is required for activation of Rac and downstream activation of MLKs, MKK7 and JNK activation on JIP1 platform in MEF cells, white adipose tissue, and muscle, but other isoforms of JIP could be essential in liver.

The complexity of JNK activation loop is also illustrated by retroinhibition of MAPK cascade by p38a in receptor-mediated cell death [41]. Indeed, p38a is activated by MKK4 and MKK3/6, and p38 then contributes inhibitory crosstalk to the JNK activation loop via inhibitory phosphorylation of TGF-beta activated kinase 1 (TAK1/TAB1), MKK3/6 and/or increased expression of DUSP (MKP) by p38a/MK2 pathway [42,43,44]. However, this regulatory pathway requires further exploration. In fact, p38 affects several alternative pathways to interfere with the JNK activation loop, such as NF-κB-mediated upregulation of antioxidant genes and 17 kDa Gadd45β (MyD118) [45,46]. Endogenous Gadd45β and MKK7 associate through direct, high-affinity contact. Gadd45β, which is not a phosphatase, inhibits MKK7 by masking the kinase domain. The association is tighter than JIP1 and thus spatially prevents MKK7 activation of JNK. Gadd45β does not inhibit MKK4, MKK3b, or ASK1 activation or phosphorylation of MKK7. Gadd45β expression is suppressed by orphan nuclear receptor small heterodimer partner (SHP), which is a transcriptional corepressor. Depletion of SHP increases Gadd45β expression and prevents sustained JNK activation and liver injury. Additionally, activation of Akt in a parallel survival pathway could activate NF-κB and inhibit MAP3K, such as MLK3 and ASK1 [47,48,49,50].

### 3.3. MAP3K—MLK2/3, ASK1, TAK1

MEKK1-4, DLK, TPL-2, TAO1/2, ASK1, MLK2/3, and TAK1 are common MAP3Ks involved in the activation pathway of JNK and p38, but the unique structure and subcellular distribution of these MAP3Ks reveal important roles in various cells and disease models. ASK1, MLK2/3, and TAK1 are widely studied and discussed in this review. ASK1 triggers cellular responses to redox stress and inflammatory cytokines [51,52] and plays important roles in innate immunity and viral infection [53]. ASK1 has the central kinase domain flanked on either side by coiled-coil domains. The N terminus of the kinase domain contains several regions with regulatory roles that bind to thioredoxin and TNF receptor-associated factors (TRAFs), which regulate the response of ASK1 to ROS and cytokines, respectively [54]. The N-terminal region of ASK1 has also been implicated in binding CIB1 to detect Ca^2+^-based stress signaling and in binding FBXO21 to trigger innate antiviral signaling [55,56]. The region C terminal to the kinase domain contains a 14-3-3 protein-binding site [57], followed by a region for constitutive oligomerization of ASK1 [58]. Under redox stress, thioredoxin dissociates and TRAF proteins associate with ASK1, which then tightly oligomerizes through its N-terminal coiled-coil (NCC) domain, promoting ASK1 activation and kinase activity via autophosphorylation of Thr845 in its kinase domain [59]. However, oxidative stress induces ubiquitination and subsequent degradation of activated ASK1. On the other hand, the deubiquitinating enzyme USP9X, which has ubiquitin-specific protease activity, interacts and antagonizes ubiquitination and subsequent degradation of activated ASK1 in H2O2-treated cells, resulting in the stabilization of activated ASK1 [60]. However, TNFAIP3 (A20), which has both ubiquitin ligase and deubiquitinase activities, inactivates ASK1 in fatty-acid-induced cells and ameliorates NASH [61]. Overexpression of A20 interacts with ASK1 and reduces stability and promotes the degradation of ASK1 through the ubiquitination process. Thus, overexpression of A20 lowers ASK1 level and preconditions cells to resist stress-induced JNK activation and cell death [62]. A20 is an acute response gene and overall effects of A20 will also depend on context and acute versus chronic disease. Contribution of A20 on JNK-SAB-ROS activation loop appears to be of minor importance. In addition to phosphorylation- and ubiquitination-mediated regulation of ASK1 in basal and response to oxidative stress, cFLIP competes for binding to TRAF binding domain of ASK1 and prevents ASK1 dimerization and activation [63]. However, ITCH promotes Lys48 ubiquitination and degradation of cFLIP. ITCH is activated by JNK [64]. JNK-ITCH-Ask1 signal activation axis does not affect TNF/GalN-induced apoptosis in contexts where SAB is deleted because depletion of SAB or MKK4/7 completely prevents TNFα-induced JNK activation and cell death, indicating requirement of MAP2K, JNK, and SAB to sustain ASK1 activation [7,12,65]. As mitochondria have thioredoxin-2, mitochondria localization of ASK1 and association with thioredoxin-2 has been proposed [66]. However, JNK activation has not been observed in mitochondria and further exploration is required. Another ASK1 regulator that has been recently identified is Caspase Recruitment Domain Protein 6 (CARD6) [67,68]. CARD6 associates with ASK1 and suppresses ASK1 phosphorylation activation and downstream JNK/p38 activation. In high-fat diet-induced fatty liver model, ASK1, MLK3, and TAK1 activation occurs. ASK1 phosphorylation is further increased by CARD6 deficiency but suppressed by CARD6 overexpression. TAK1 phosphorylation is not affected by CARD6, indicating selectivity of regulation. TAK1 is phosphorylated and activated via TLR/IL1 receptor and TRAFs [69]. Phosphorylated TAK1 activates IKK and MAP2K, leading to activation of NF-κB and JNK, respectively. Hepatocyte-specific deletion of TAK1 causes spontaneous hepatocyte death, suggesting hepatoprotective role of TAK1 [70], although further exploration is required.

MLK2/3 are redundant MAP3Ks regulated by small GTPases CDC42 and RAC1 [71]. Recently, JIP1 has been identified as a platform for interaction and signal integration of SRC tyrosine kinase, RAC GTPase, and MLKs for activation of MKK7 and JNK in free fatty acid-induced activation model [19]. As JIP is a JNK-specific scaffold protein, the pathway selectively activates JNK but not p38 in free fatty acid-induced stress in MEF cells and diet-induced mouse models. The role of ASK1 was not examined in this free fatty acid-induced stress in MEF cell model. Indeed, JIP3/4 interacts with ASK1 but cannot mediate JNK activation [72]. The importance of JIP/MAP3K/MAP2K/MAPK signaling pathway in death receptor (TNF)-mediated MAP kinase activation requires further exploration. 

### 3.4. Scaffold Protein—SAB

SAB is a mitochondrial outer membrane protein with N-terminal SH3 domain binding site, one membrane spanning domain, and two D-motif (KIM) on C-terminus [11]. The topology of SAB makes it unique in JNK-mediated signal transduction to mitochondria. The N-terminal of SAB, including SH3-domain-binding site, is in the mitochondria intermembrane space, and C-terminal of SAB with KIM motif is facing the cytoplasm [12]. SAB is the only JNK docking site on mitochondria. The depletion of SAB completely prevents JNK translocation to mitochondria [7,12]. Both JNK and p38 can phosphorylate SAB in cell-free system [73], but in vivo evidence is lacking. The deletion of SAB does not inhibit p38 association with mitochondria [65]. The SH3-domain-binding site of SAB is largely unexplored. The recent identification of the SAB homolog RAB-11-interacting protein-1 (REI-1), a guanine nucleotide exchange factor (GEF) that is homologous to the N-terminal of mammalian SAB, is associated with Rab11 of *C. elegans* [74]. Thus, further explorations are required to examine the role of RAB GTPase in JNK-SAB-ROS activation loop. As PTPN6 dissociates from SAB when JNK interacts with and phosphorylates SAB, there could be possible regulation of the PTPN6 dissociation from SAB. Therefore, RAB like GTPase could be associated with intramitochondrial portion of SAB and might participate in regulation of the JNK-SAB-ROS activation loop. There are other GTPases that have been identified as facing into the intermitochondrial membrane space, such as OPA1, which is regulated by SIRT3 [75], but the association with SAB is not known.

### 3.5. Regulation of SAB Expression

We have recently begun to address the role of the regulation of SAB expression. We have initially gained insight into this area through overexpression of SAB as well as through exploration of sex differences in susceptibility to acute liver injury in mouse models. We expressed Adeno-SAB in liver-specific SAB knockout mice using increasing doses of adenovirus and found that increasing levels of SAB expression led to increasing susceptibility to injury from a fixed nonlethal dose of APAP. This not only demonstrates that SAB restores susceptibility to liver injury in SAB knockout mice but also that the level of SAB expression determined the severity of liver injury. Furthermore, inducible hepatocyte knockout of JNK1 and 2 (AAV8-TBG-CRE) in *JNK1/2^fl/fl^* mice markedly protected against APAP injury, which was not increased with concomitant SAB overexpression. This indicates that JNK is required for enhanced susceptibility to APAP injury due to SAB overexpression and that there is no other pathway (other than JNK) for the participation of SAB in the injury process. Furthermore, JNK1/2 deletion did not affect SAB basal expression and vice versa.

It is well known that female mice are very resistant to APAP toxicity in vivo. We confirmed this and found that the resistance applied to TNF/GalN in vivo as well as palmitic acid-induced lipoapoptosis in primary mouse hepatocytes. In all these models, female littermates exhibited markedly decreased levels of sustained JNK activation. This led us to examine SAB expression, which was found to be markedly decreased in females (only 15% of male liver mitochondrial level of SAB). Similar sex difference in SAB expression was observed in normal human liver. We then identified post-transcriptional regulation of SAB expression (repression in females) involving a pathway from estrogen receptor-α to p53 (higher expression in female mouse and human liver) to p53-mediated expression of miR34a-5p, which targets the SAB mRNA coding region, thus repressing SAB expression and decreasing susceptibility to liver injury (abstract, manuscript in preparation). There is currently no information on the transcriptional regulation of SAB expression, and this is an important area we are exploring. 

## 4. Perspectives on the Intervention of the JNK Activation Loop

JNK-SAB-ROS activation loop is an important cell death-promoting pathway in apoptosis and mitochondrial permeability transition pore (MPT)-regulated necrosis (in the context of acetaminophen hepatotoxicity). The pathway is modulated by several parallel survival pathways through crosstalk and negative regulatory feedback. Any adaptation or mechanism changing the balance of survival and death pathways will partially interfere with the JNK-SAB-ROS pathway directly or indirectly and the cell death outcome. Thus, targeting molecules in JNK-SAB-ROS activation loop is a promising strategy to promote cell death, such as in cancer cells [76,77], and to prevent cell death, such as in hepatotoxicity [7,12,65], liver and kidney injury in septic shock, and ischemia/reperfusion injury in heart and brain [78,79,80,81]. A selective ASK1 inhibitor, selonsertib (GS-4997), has recently been tested as therapy for NASH in a phase 2 clinical trial (NCT02466516), and patient outcomes were encouraging. Targeting the pivotal role of SAB in JNK activation offers particular promise. Blocking the binding of P-JNK to SAB using KIM1 peptides can be selectively achieved without directly blocking the kinase activity of JNK [8,9,82]. Thus, identification of selective small molecule inhibitors of the binding of P-JNK to SAB seems feasible. Modulating expression of SAB (increase or decrease) may be possible through modulation of the factors that control transcriptional and post-translational regulation (e.g., transcription factors and noncoding RNA that target SAB expression). In addition, antisense oligonucleotides that are cell-type- or organ-specific are being developed to lower SAB expression. These approaches for modification of SAB expression appear to offer the most promise in chronic diseases where sustained JNK activation affects metabolism.

## Abbreviations

AKT	AKT serine/threonine kinase 1
APAP	acetaminophen
ASK1	apoptosis signal-regulating kinase 1
ATF2	activating transcription factor 2
ATP	adenosine triphosphate
CDC42	cell division cycle 42
cFLIP	CASP8 and FADD like apoptosis regulator
c-JUN	Jun proto-oncogene
DOK4	downstream of tyrosine kinase/docking protein 4
DUSP	dual-specificity phosphatase
ER	endoplasmic reticulum
ERK	extracellular signal-regulated kinase
GalN	*N*-acetyl-galactosamine
ITCH	Itchy E3 ubiquitin protein ligase
JIP	JNK-interacting protein
JNK	c-Jun N-terminal kinases
KIM	kinase interaction motif
KO	knock out
LPS	lipopolysaccharide
MAPK	mitogen-activated protein kinase
MAP2K	mitogen-activated protein kinase kinase
MAP3K	mitogen-activated protein kinase kinase kinase
MKK4	MAPK kinase 4
MKP	mitogen-activated protein kinase phosphatase
MLK	mixed lineage kinase
MPT	mitochondrial permeability transition
NASH	nonalcoholic steatohepatitis
NCOR	nuclear receptor corepressor
NF-κB	nuclear factor kappa-light-chain-enhancer of activated B cells
P-JNK	phosphoactivated JNK
RAC	Rac family small GTPase 1
ROS	reactive oxygen species
SAB (Sh3bp5)	SH3-domain binding protein 5
SH3	SRC Homology 3 Domain
SHP1	SH2 phosphatase 1
SRC	SRC proto-oncogene non-receptor tyrosine kinase
TAK1	TGF-beta activated kinase 1
TNF	tumor necrosis factor

## References

[1] Bogoyevitch M.A., Kobe B. (2006). Uses for JNK: The many and varied substrates of the c-Jun N-terminal kinases. Microbiol. Mol. Biol. Rev..

[2] Weston C.R., Davis R.J. (2007). The JNK signal transduction pathway. Curr. Opin. Cell. Biol..

[3] Win S., Than T.A., Zhang J., Oo C., Min R.W.M., Kaplowitz N. (2018). New insights into the role and mechanism of c-Jun-N-terminal kinase signaling in the pathobiology of liver diseases. Hepatology.

[4] Tobiume K., Matsuzawa A., Takahashi T., Nishitoh H., Morita K., Takeda K., Minowa O., Miyazono K., Noda T., Ichijo H. (2001). ASK1 is required for sustained activations of JNK/p38 MAP kinases and apoptosis. EMBO Rep..

[5] Gunawan B.K., Liu Z.X., Han D., Hanawa N., Gaarde W.A., Kaplowitz N. (2006). c-Jun N-terminal kinase plays a major role in murine acetaminophen hepatotoxicity. Gastroenterology.

[6] Hanawa N., Shinohara M., Saberi B., Gaarde W.A., Han D., Kaplowitz N. (2008). Role of JNK translocation to mitochondria leading to inhibition of mitochondria bioenergetics in acetaminophen-induced liver injury. J. Biol. Chem..

[7] Win S., Than T.A., Han D., Petrovic L.M., Kaplowitz N. (2011). c-Jun N-terminal kinase (JNK)-dependent acute liver injury from acetaminophen or tumor necrosis factor (TNF) requires mitochondrial Sab protein expression in mice. J. Biol. Chem..

[8] Win S., Than T.A., Fernandez-Checa J.C., Kaplowitz N. (2014). JNK interaction with Sab mediates ER stress induced inhibition of mitochondrial respiration and cell death. Cell Death Dis..

[9] Win S., Than T.A., Le B.H., García-Ruiz C., Fernandez-Checa J.C., Kaplowitz N. (2015). Sab (Sh3bp5) dependence of JNK mediated inhibition of mitochondrial respiration in palmitic acid induced hepatocyte lipotoxicity. J. Hepatol..

[10] Kamata H., Honda S., Maeda S., Chang L., Hirata H., Karin M. (2005). Reactive oxygen species promote TNFalpha-induced death and sustained JNK activation by inhibiting MAP kinase phosphatases. Cell.

[11] Wiltshire C., Matsushita M., Tsukada S., Gillespie D.A., May G.H. (2002). A new c-Jun N-terminal kinase (JNK)-interacting protein, Sab (SH3BP5), associates with mitochondria. Biochem. J..

[12] Win S., Than T.A., Min R.W., Aghajan M., Kaplowitz N. (2016). c-Jun N-terminal kinase mediates mouse liver injury through a novel Sab (SH3BP5)-dependent pathway leading to inactivation of intramitochondrial Src. Hepatology.

[13] Liu Q., Rehman H., Krishnasamy Y., Schnellmann R.G., Lemasters J.J., Zhong Z. (2015). Improvement of liver injury and survival by JNK2 and iNOS deficiency in liver transplants from cardiac death mice. J. Hepatol..

[14] Huo Y., Win S., Than T.A., Yin S., Ye M., Hu H., Kaplowitz N. (2017). Antcin H Protects Against Acute Liver Injury Through Disruption of the Interaction of c-Jun-N-Terminal Kinase with Mitochondria. Antioxid. Redox Signal..

[15] Itoh S., Lemay S., Osawa M., Che W., Duan Y., Tompkins A., Brookes P.S., Sheu S.S., Abe J. (2005). Mitochondrial Dok-4 recruits Src kinase and regulates NF-kappaB activation in endothelial cells. J. Biol. Chem..

[16] Mishra P., Günther S. (2018). New insights into the structural dynamics of the kinase JNK3. Sci. Rep..

[17] Laughlin J.D., Nwachukwu J.C., Figuera-Losada M., Cherry L., Nettles K.W., LoGrasso P.V. (2012). Structural mechanisms of allostery and autoinhibition in JNK family kinases. Structure.

[18] Jaeschke A., Czech M.P., Davis R.J. (2004). An essential role of the JIP1 scaffold protein for JNK activation in adipose tissue. Genes Dev..

[19] Morel C., Standen C.L., Jung D.Y., Gray S., Ong H., Flavell R.A., Kim J.K., Davis R.J. (2010). Requirement of JIP1-mediated c-Jun N-terminal kinase activation for obesity-induced insulin resistance. Mol. Cell. Biol..

[20] Kant S., Standen C.L., Morel C., Jung D.Y., Kim J.K., Swat W., Flavell R.A., Davis R.J. (2017). A Protein Scaffold Coordinates SRC-Mediated JNK Activation in Response to Metabolic Stress. Cell Rep..

[21] Chambers J.W., LoGrasso P.V. (2011). Mitochondrial c-Jun N-terminal kinase (JNK) signaling initiates physiological changes resulting in amplification of reactive oxygen species generation. J. Biol. Chem..

[22] Fuchs S.Y., Adler V., Pincus M.R., Ronai Z. (1998). MEKK1/JNK signaling stabilizes and activates p53. Proc. Natl. Acad. Sci. USA.

[23] Cargnello M., Roux P.P. (2011). Activation and function of the MAPKs and their substrates, the MAPK-activated protein kinases. Microbiol. Mol. Biol. Rev..

[24] Sabio G., Davis R.J. (2014). TNF and MAP kinase signaling pathways. Semin. Immunol..

[25] Pérez S., Rius-Pérez S., Tormos A.M., Finamor I., Nebreda Á.R., Taléns-Visconti R., Sastre J. (2018). Age-dependent regulation of antioxidant genes by p38α MAPK in the liver. Redox Biol..

[26] Weber H.O., Ludwig R.L., Morrison D., Kotlyarov A., Gaestel M., Vousden K.H. (2005). HDM2 phosphorylation by MAPKAP kinase 2. Oncogene.

[27] Malmlöf M., Roudier E., Högberg J., Stenius U. (2007). MEK-ERK-mediated phosphorylation of Mdm2 at Ser-166 in hepatocytes. Mdm2 is activated in response to inhibited Akt signaling. J. Biol. Chem..

[28] Alarcon-Vargas D., Ronai Z. (2002). p53-Mdm2--the affair that never ends. Carcinogenesis.

[29] Carr M.I., Jones S.N. (2016). Regulation of the Mdm2-p53 signaling axis in the DNA damage response and tumorigenesis. Transl. Cancer Res..

[30] Huo Y., Yin S., Yan M., Win S., Aung Than T., Aghajan M., Hu H., Kaplowitz N. (2017). Protective role of p53 in acetaminophen hepatotoxicity. Free Radic Biol. Med..

[31] Huang S., Shu L., Dilling M.B., Easton J., Harwood F.C., Ichijo H., Houghton P.J. (2003). Sustained activation of the JNK cascade and rapamycin-induced apoptosis are suppressed by p53/p21(Cip1). Mol. Cell.

[32] Huang C.Y., Tan T.H. (2012). DUSPs, to MAP kinases and beyond. Cell Biosci..

[33] Sarikhani M., Mishra S., Desingu P.A., Kotyada C., Wolfgeher D., Gupta M.P., Singh M., Sundaresan N.R. (2018). SIRT2 regulates oxidative stress-induced cell death through deacetylation of c-Jun NH2-terminal kinase. Cell Death Differ..

[34] Villalba J.M., Alcaín F.J. (2012). Sirtuin activators and inhibitors. Biofactors.

[35] Cui X., Chen Q., Dong Z., Xu L., Lu T., Li D., Zhang J., Zhang M., Xia Q. (2016). Inactivation of Sirt1 in mouse livers protects against endotoxemic liver injury by acetylating and activating NF-κB. Cell Death Dis..

[36] Singh C.K., Chhabra G., Ndiaye M.A., Garcia-Peterson L.M., Mack N.J., Ahmad N. (2018). The Role of Sirtuins in Antioxidant and Redox Signaling. Antioxid. Redox Signal..

[37] Ho D.T., Bardwell A.J., Abdollahi M., Bardwell L. (2003). A docking site in MKK4 mediates high affinity binding to JNK MAPKs and competes with similar docking sites in JNK substrates. J. Biol. Chem..

[38] Kragelj J., Palencia A., Nanao M.H., Maurin D., Bouvignies G., Blackledge M., Jensen M.R. (2015). Structure and dynamics of the MKK7-JNK signaling complex. Proc. Natl. Acad. Sci. USA.

[39] Sun Y., Li T.Y., Song L., Zhang C., Li J., Lin Z.Z., Lin S.C., Lin S.Y. (2018). Liver-specific deficiency of unc-51 like kinase 1 and 2 protects mice from acetaminophen-induced liver injury. Hepatology.

[40] Zeke A., Misheva M., Reményi A., Bogoyevitch M.A. (2016). JNK Signaling: Regulation and Functions Based on Complex Protein-Protein Partnerships. Microbiol. Mol. Biol. Rev..

[41] Heinrichsdorff J., Luedde T., Perdiguero E., Nebreda A.R., Pasparakis M. (2008). p38 alpha MAPK inhibits JNK activation and collaborates with IkappaB kinase 2 to prevent endotoxin-induced liver failure. EMBO Rep..

[42] Zarubin T., Han J. (2005). Activation and signaling of the p38 MAP kinase pathway. Cell Res..

[43] Girnius N., Davis R.J. (2016). TNFα-Mediated Cytotoxic Responses to IAP Inhibition Are Limited by the p38α MAPK Pathway. Cancer Cell.

[44] Lalaoui N., Hänggi K., Brumatti G., Chau D., Nguyen N.N., Vasilikos L., Spilgies L.M., Heckmann D.A., Ma C., Ghisi M. (2016). Targeting p38 or MK2 Enhances the Anti-Leukemic Activity of Smac-Mimetics. Cancer Cell.

[45] De Smaele E., Zazzeroni F., Papa S., Nguyen D.U., Jin R., Jones J., Cong R., Franzoso G. (2001). Induction of gadd45beta by NF-kappaB downregulates pro-apoptotic JNK signaling. Nature.

[46] Papa S., Zazzeroni F., Bubici C., Jayawardena S., Alvarez K., Matsuda S., Nguyen D.U., Pham C.G., Nelsbach A.H., Melis T. (2004). Gadd45 beta mediates the NF-kappa B suppression of JNK signaling by targeting MKK7/JNKK2. Nat. Cell Biol..

[47] Bai D., Ueno L., Vogt P.K. (2009). Akt-mediated regulation of NFkappaB and the essentialness of NFkappaB for the oncogenicity of PI3K and Akt. Int. J. Cancer.

[48] Barthwal M.K., Sathyanarayana P., Kundu C.N., Rana B., Pradeep A., Sharma C., Woodgett J.R., Rana A. (2003). Negative regulation of mixed lineage kinase 3 by protein kinase B/AKT leads to cell survival. J. Biol. Chem..

[49] Lin Z., Liu T., Kamp D.W., Wang Y., He H., Zhou X., Li D., Yang L., Zhao B., Liu G. (2014). AKT/mTOR and c-Jun N-terminal kinase signaling pathways are required for chrysotile asbestos-induced autophagy. Free Radic. Biol. Med..

[50] Song J.J., Lee Y.J. (2005). Dissociation of Akt1 from its negative regulator JIP1 is mediated through the ASK1-MEK-JNK signal transduction pathway during metabolic oxidative stress: A negative feedback loop. J. Cell Biol..

[51] Soga M., Matsuzawa A., Ichijo H. (2012). Oxidative Stress-Induced Diseases via the ASK1 Signaling Pathway. Int. J. Cell Biol..

[52] Ichijo H., Nishida E., Irie K., ten Dijke P., Saitoh M., Moriguchi T., Takagi M., Matsumoto K., Miyazono K., Gotoh Y. (1997). Induction of apoptosis by ASK1, a mammalian MAPKKK that activates SAPK/JNK and p38 signaling pathways. Science.

[53] Matsuzawa A., Saegusa K., Noguchi T., Sadamitsu C., Nishitoh H., Nagai S., Koyasu S., Matsumoto K., Takeda K., Ichijo H. (2005). ROS-dependent activation of the TRAF6-ASK1-p38 pathway is selectively required for TLR4-mediated innate immunity. Nat. Immunol..

[54] Weijman J.F., Kumar A., Jamieson S.A., King C.M., Caradoc-Davies T.T., Ledgerwood E.C., Murphy J.M., Mace P.D. (2017). Structural basis of autoregulatory scaffolding by apoptosis signal-regulating kinase 1. Proc. Natl. Acad. Sci. USA.

[55] Yoon K.W., Cho J.H., Lee J.K., Kang Y.H., Chae J.S., Kim Y.M., Kim J., Kim E.K., Kim S.E., Baik J.H. (2009). CIB1 functions as a Ca(2+)-sensitive modulator of stress-induced signaling by targeting ASK1. Proc. Natl. Acad. Sci. USA.

[56] Yu Z., Chen T., Li X., Yang M., Tang S., Zhu X., Gu Y., Su X., Xia M., Li W. (2016). Lys29-linkage of ASK1 by Skp1-Cullin 1-Fbxo21 ubiquitin ligase complex is required for antiviral innate response. Elife.

[57] Cockrell L.M., Puckett M.C., Goldman E.H., Khuri F.R., Fu H. (2010). Dual engagement of 14-3-3 proteins controls signal relay from ASK2 to the ASK1 signalosome. Oncogene.

[58] Kawarazaki Y., Ichijo H., Naguro I. (2014). Apoptosis signal-regulating kinase 1 as a therapeutic target. Expert Opin. Ther. Targets.

[59] Cebula M., Schmidt E.E., Arnér E.S. (2015). TrxR1 as a potent regulator of the Nrf2-Keap1 response system. Antioxid. Redox Signal..

[60] Nagai H., Noguchi T., Homma K., Katagiri K., Takeda K., Matsuzawa A., Ichijo H. (2009). Ubiquitin-like sequence in ASK1 plays critical roles in the recognition and stabilization by USP9X and oxidative stress-induced cell death. Mol. Cell.

[61] Zhang P., Wang P.X., Zhao L.P., Zhang X., Ji Y.X., Zhang X.J., Fang C., Lu Y.X., Yang X., Gao M.M. (2018). The deubiquitinating enzyme TNFAIP3 mediates inactivation of hepatic ASK1 and ameliorates nonalcoholic steatohepatitis. Nat. Med..

[62] Won M., Park K.A., Byun H.S., Sohn K.C., Kim Y.R., Jeon J., Hong J.H., Park J., Seok J.H., Kim J.M. (2010). Novel anti-apoptotic mechanism of A20 through targeting ASK1 to suppress TNF-induced JNK activation. Cell Death Differ..

[63] Wang P.X., Ji Y.X., Zhang X.J., Zhao L.P., Yan Z.Z., Zhang P., Shen L.J., Yang X., Fang J., Tian S. (2017). Targeting CASP8 and FADD-like apoptosis regulator ameliorates nonalcoholic steatohepatitis in mice and nonhuman primates. Nat. Med..

[64] Chang L., Kamata H., Solinas G., Luo J.L., Maeda S., Venuprasad K., Liu Y.C., Karin M. (2006). The E3 ubiquitin ligase itch couples JNK activation to TNFalpha-induced cell death by inducing c-FLIP(L.) turnover. Cell.

[65] Zhang J., Min R.W.M., Le K., Zhou S., Aghajan M., Than T.A., Win S., Kaplowitz N. (2017). The role of MAP2 kinases and p38 kinase in acute murine liver injury models. Cell Death Dis..

[66] Zhang R., Al-Lamki R., Bai L., Streb J.W., Miano J.M., Bradley J., Min W. (2004). Thioredoxin-2 inhibits mitochondria-located ASK1-mediated apoptosis in a JNK-independent manner. Circ. Res..

[67] Qin J.J., Mao W., Wang X., Sun P., Cheng D., Tian S., Zhu X.Y., Yang L., Huang Z., Li H. (2018). Caspase recruitment domain 6 protects against hepatic ischemia/reperfusion injury by suppressing ASK1. J. Hepatol..

[68] Sun P., Zeng Q., Cheng D., Zhang K., Zheng J., Liu Y., Yuan Y.F., Tang Y.D. (2018). Caspase Recruitment Domain Protein 6 protects against hepatic steatosis and insulin resistance by suppressing Ask1. Hepatology.

[69] Takaesu G., Ninomiya-Tsuji J., Kishida S., Li X., Stark G.R., Matsumoto K. (2001). Interleukin-1 (IL-1) receptor-associated kinase leads to activation of TAK1 by inducing TAB2 translocation in the IL-1 signaling pathway. Mol. Cell. Biol..

[70] Malato Y., Willenbring H. (2010). The MAP3K TAK1: A chock block to liver cancer formation. Hepatology.

[71] Sharma M., Urano F., Jaeschke A. (2012). Cdc42 and Rac1 are major contributors to the saturated fatty acid-stimulated JNK pathway in hepatocytes. J. Hepatol..

[72] Kelkar N., Standen C.L., Davis R.J. (2005). Role of the JIP4 scaffold protein in the regulation of mitogen-activated protein kinase signaling pathways. Mol. Cell. Biol..

[73] Court N.W., Kuo I., Quigley O., Bogoyevitch M.A. (2004). Phosphorylation of the mitochondrial protein Sab by stress-activated protein kinase 3. Biochem. Biophys. Res. Commun..

[74] Sakaguchi A., Sato M., Sato K., Gengyo-Ando K., Yorimitsu T., Nakai J., Hara T., Sato K., Sato K. (2015). REI-1 Is a Guanine Nucleotide Exchange Factor Regulating RAB-11 Localization and Function in C. elegans Embryos. Dev. Cell.

[75] Samant S.A., Zhang H.J., Hong Z., Pillai V.B., Sundaresan N.R., Wolfgeher D., Archer S.L., Chan D.C., Gupta M.P. (2014). SIRT3 deacetylates and activates OPA1 to regulate mitochondrial dynamics during stress. Mol. Cell. Biol..

[76] Paudel I., Hernandez S.M., Portalatin G.M., Chambers T.P., Chambers J.W. (2018). Sab Concentrations Indicate Chemotherapeutic Susceptibility in Ovarian Cancer Cell Lines. Biochem. J..

[77] Chambers T.P., Portalatin G.M., Paudel I., Robbins C.J., Chambers J.W. (2015). Sub-chronic administration of LY294002 sensitizes cervical cancer cells to chemotherapy by enhancing mitochondrial JNK signaling. Biochem. Biophys. Res. Commun..

[78] Chambers J.W., Pachori A., Howard S., Iqbal S., LoGrasso P.V. (2013). Inhibition of JNK mitochondrial localization and signaling is protective against ischemia/reperfusion injury in rats. J. Biol. Chem..

[79] Chambers T.P., Santiesteban L., Gomez D., Chambers J.W. (2017). Sab mediates mitochondrial dysfunction involved in imatinib mesylate-induced cardiotoxicity. Toxicology.

[80] Chambers J.W., Howard S., LoGrasso P.V. (2013). Blocking c-Jun N-terminal kinase (JNK) translocation to the mitochondria prevents 6-hydroxydopamine-induced toxicity in vitro and in vivo. J. Biol. Chem..

[81] Chambers J.W., Pachori A., Howard S., Ganno M., Hansen D.J., Kamenecka T., Song X., Duckett D., Chen W., Ling Y.Y. (2011). Small Molecule c-jun-N-terminal Kinase (JNK) Inhibitors Protect Dopaminergic Neurons in a Model of Parkinson’s Disease. ACS Chem. Neurosci..

[82] Chambers J.W., Cherry L., Laughlin J.D., Figuera-Losada M., Lograsso P.V. (2011). Selective inhibition of mitochondrial JNK signaling achieved using peptide mimicry of the Sab kinase interacting motif-1 (KIM1). ACS Chem. Biol..

